# Exploring the Influence of Insulin Resistance on Arterial Stiffness in Healthy Adults: From the Metabolic and Cardiovascular Health Insights of the EVasCu Study

**DOI:** 10.3390/nu16060791

**Published:** 2024-03-11

**Authors:** Carlos Pascual-Morena, Iván Cavero-Redondo, Irene Martínez-García, Eva Rodríguez-Gutiérrez, Maribel Lucerón-Lucas-Torres, Nerea Moreno-Herráiz, Valentina Díaz-Goñi, Alicia Saz-Lara

**Affiliations:** 1 Health and Social Research Center, Universidad de Castilla-La Mancha, 16071 Cuenca, Spain; carlos.pascual@uclm.es (C.P.-M.); irene.mgarcia@uclm.es (I.M.-G.); eva.rodriguez@uclm.es (E.R.-G.); mariaisabel.luceron@uclm.es (M.L.-L.-T.); nerea.moreno@uclm.es (N.M.-H.); mvalentina.diaz@alu.uclm.es (V.D.-G.); alicia.delsaz@uclm.es (A.S.-L.); 2 Faculty of Nursing, Universidad de Castilla-La Mancha, 02006 Albacete, Spain; 3 Facultad de Ciencias de la Salud, Universidad Autónoma de Chile, Talca 3460000, Chile

**Keywords:** insulin resistance, vascular stiffness, pulse wave analysis, cardiovascular health, endocrinology, metabolism, epidemiology, adult

## Abstract

Previous evidence associates insulin resistance with arterial stiffness in various pathologies, yet limited reports exist in healthy adults. Therefore, this study aims to estimate the association between insulin resistance and arterial stiffness in healthy adults. The cross-sectional EVasCu study enrolled 390 participants (42.05 ± 13.15 years). ANCOVAs, unadjusted (model 1) and adjusted (model 2), explored the association between arterial stiffness markers (aortic Pulse Wave Velocity [aPWV], Augmentation Index [AIx@75] and Cardio-Ankle Vascular Index [CAVI]), and insulin resistance markers (Homeostasis Model Assessment of Insulin Resistance [HOMA-IR], Quantitative Insulin Sensitivity Check Index [QUICKI] and Triglycerides-Glucose [TyG]). In model 1, all insulin resistance markers were associated with aPWV, HOMA-IR and QUICKI were associated with AIx@75, and the TyG index was associated with CAVI. In model 2, HOMA-IR and QUICKI increased aPWV by 0.179 and 0.156 m/s (*p* = 0.001 and *p* = 0.011), and AIx@75 by 4.17 and 5.39% (*p* = 0.009 and *p* = 0.003). The EVasCu study offers valuable insights into the relationship between insulin resistance and arterial stiffness in healthy adults, providing a deeper understanding of metabolic and cardiovascular health. By examining this influence, we embark on an intriguing exploration of how these factors interplay in the human body.

## 1. Introduction

Insulin plays an important role in regulating metabolism, from glucose uptake and metabolism by cells, to ion transport across cell membranes, protein synthesis and gene transcription. Individuals with insulin resistance are those who, at a given plasma insulin concentration, are unable to uptake and metabolise as much glucose as they should [1]. Insulin resistance has a prevalence of approximately 15.5% in Europeans and even higher for Americans [2,3] and has risk factors such as genetics, diet, dyslipidaemia or obesity [1,4]. Insulin resistance is not only a precursor to type 2 diabetes mellitus [5], but also increases the risk of cardiovascular and cerebrovascular events [5,6]. The most commonly used measure of insulin resistance is the Homeostatic Model Assessment for Insulin Resistance (HOMA-IR), although there are others such as the Quantitative Insulin Sensitivity Check Index (QUICKI) and the Triglycerides-Glucose Index (TyG Index) [7].

Obesity, arterial hypertension and arterial stiffness are other risk factors for cardiovascular events [8]. Arterial stiffness has been proposed as an independent risk factor in the prognostic risk classification of peripheral arterial, coronary and cerebral disease [9]. In addition, insulin resistance can increase arterial stiffness. Under physiological conditions, insulin promotes vascular relaxation and nitric oxide production with a vasodilatory effect. However, in individuals with insulin resistance, insulin action is reduced and may even cause vasoconstriction due to increased endothelin production, decreased nitric oxide synthesis and smooth muscle remodelling, including hypertrophy and hyperplasia [10,11]. The latter could be because chronic exposure to hyperglycaemia induces the proliferation of vascular smooth muscle cells and advanced glycation products, and insulin resistance induces the synthesis of collagen and the expression of proinflammatory genes [12].

Different techniques and approaches can be used to measure arterial stiffness. The most common are noninvasive techniques, such as applanation tonometry and oscillometric techniques, which are quick, simple and nontraumatic. Applanation tonometry and oscillometric techniques can be used to estimate carotid-femoral Pulse Wave Velocity (cfPWV) and aortic Pulse Wave Velocity (aPWV), respectively, with a cut-off of 10 meters per second (m/s) for determining possible arterial stiffness [13,14]. The Augmentation Index value adjusted to a heart rate of 75 beats per minute (AIx@75) is another parameter of arterial stiffness. AIx@75 is estimated from the central arterial pulse wave, which consists of a forward wave generated by ventricular ejection and a backwards wave that depends on the elastic properties of the arteries, transmission velocity and distance. Previous evidence suggests that values above 40% are predictive of cardiovascular events [15,16]. Finally, the Cardio-Ankle Vascular Index (CAVI) is another key parameter of arterial stiffness that can be estimated independently of blood pressure, the most common cut-off being 8 m/s [17,18]. 

Insulin resistance and other associated risk factors can increase arterial stiffness. However, most studies have been conducted in populations with pathologies (e.g., diabetes mellitus), with exceptions [19]. Therefore, the aim of this study was to estimate the association between insulin resistance, as measured by the HOMA-IR, QUICKI and TyG index, and arterial stiffness, as measured by aPWV, AIx@75 and CAVI, in a population without diagnosed pathologies whose treatment could modify arterial stiffness.

## 2. Materials and Methods

### 2.1. Study Design

The EVasCu study was a cross-sectional study conducted in the province of Cuenca (Spain) designed to assess the validity of a model of early vascular ageing as an index of cardiovascular risk in healthy adults [20]. The study protocol followed the Declaration of Helsinki and was previously approved by the Clinical Research Ethics Committee of the Cuenca Health Area (REG: 2022/PI2022). This study followed the Strengthening the Reporting of Observational Studies in Epidemiology (STROBE) statement.

### 2.2. Study Sample

A total of 390 participants were enrolled in the EVasCu study from June to December 2022, without previously diagnosed conditions that could significantly modify arterial stiffness, including diabetes mellitus, dyslipidaemia and arterial hypertension. The purpose and procedure of the study were explained to the participants, and they signed an informed consent form. Once all the results had been obtained, they were given a report validated by a doctor, with recommendations if necessary.

### 2.3. Dependent Variables

As continuous dependent variables were included, aPWV, AIx@75 and CAVI were included. Mobil-O-Graph^®^ (IEM GmbH) was used to measure aPWV and AIx75. This PWV is an estimate of aPWV, a parameter of central arterial stiffness. VaSera (FUKUDA-DENSHI) was used to measure CAVI. The aPWV and CAVI are estimated in m/s, while the AIx@75 is estimated as a percentage (%).

### 2.4. Independent Variables

Blood samples were taken between 8 and 9 am after a 12 h fast. Blood glucose (mg/dL), insulin (uU/L) and triglycerides (mg/dL) were measured using the Roche Diagnostics Cobas 8000 system (Hospital Virgen de la Luz, Cuenca). These parameters were used to estimate the variables of interest and to calculate the HOMA-IR, QUICKI and TyG index [21,22,23]. A cut-off of 2.05 was used for HOMA-IR, 2.33 for QUICKI and 8.3 for TyG index, based on Caucasian populations [23,24,25].

### 2.5. Covariates

Age, education level and smoking were reported by direct question. For education, responses were categorised into six levels: illiterate, no education, primary school, secondary school, high school and university degree. For smoking, responses were categorised into four levels: ex-smoker < 1 year, ex-smoker 1–5 years, ex-smoker > 5 years and nonsmoker.

Weight and height were measured twice and averaged for analysis using a scale (Seca^®^ 861; Vogel & Halke, Hamburg, Germany) and a wall stadiometer (Seca^®^ 222; Vogel & Halke, Hamburg, Germany), respectively. Body mass index (BMI) was calculated as weight (kg)/height (m^2^), and cut-off points of 18.5, 25.0 and 30.0 were used to classify participants as underweight, normal weight, overweight and obese [26].

Levels of total cholesterol, low-density lipoprotein cholesterol (LDL-C), high-density lipoprotein cholesterol (HDL-C) and triglycerides were measured using the same instruments as for glucose and insulin.

Blood pressure was measured in duplicate using the Omron^®^ M5-I monitor (Omron Healthcare UK Ltd., Milton Keynes, UK) after the participant had rested for 5 minutes. Mean arterial pressure (MAP) was estimated as MAP = ((systolic pressure) + (diastolic pressure × 2))/3.

### 2.6. Statistical Analysis

Normal probability plots and the Kolmogorov-Smirnov test were used to check the normality of the distribution of continuous variables. Descriptive variables were presented as the means and standard deviations (SD) or proportions (%), as appropriate. Descriptive data were presented for the total sample and stratified by sex, and Student’s *t* test for independent samples was used to determine whether there were statistically significant differences between variables according to sex.

First, linear regressions between insulin resistance and arterial stiffness were performed and presented in tables with slope, Pearson correlation coefficient and *p* value. Student’s *t* test for independent samples and analysis of variance (ANOVA) were performed between the parameters of arterial stiffness and the presence of insulin resistance, determined by HOMA-IR, QUICKI and TyG index. Analysis of covariance (ANCOVA) was then performed adjusting for all covariates, including educational level, smoking status and BMI category (as categorical covariates) and age, total cholesterol, LDL-C, HDL-C, triglyceride levels and MAP (as continuous covariates). Student’s *t* test and ANCOVAs were performed for the total sample and stratified by sex.

All statistical analyses were performed using IBM SPSS 28 (SPSS Inc., Chicago, IL, USA).

## 3. Results

### 3.1. Characteristics of Study Participants

The study included 390 participants with a mean age of 42.05 years (SD = 13.15). Of the total sample, 246 (63.1%) were women. There were no statistically significant differences between the sexes for educational level, smoking, fasting insulin, HOMA-IR, QUICKI, total cholesterol and LDL-C, while the values for BMI, fasting blood glucose, and TyG index were higher in men than in women, and HDL-C was higher in women than in men. Baseline sample characteristics are described in Table 1.

The three markers of insulin resistance were statistically significantly associated with each other, especially HOMA-IR with QUICKI (R = 0.807, *p* < 0.001) (Supplementary Table S1). Similarly, the three markers of arterial stiffness were statistically significantly associated with each other, especially aPWV with CAVI (R = 0.627, *p* < 0.001) (Supplementary Table S2).

### 3.2. Association between Insulin Resistance and Arterial Stiffness

Table 2 summarises the linear regressions between insulin resistance and arterial stiffness. Increased HOMA-IR and TyG index and decreased QUICKI were associated with increased aPWV and AIx@75. For CAVI, only the TyG index in the total population and QUICKI in men reached statistical significance.

Table 3 summarises aPWV, AIx@75 and CAVI according to the insulin resistance estimated by HOMA-IR, QUICKI and TyG indexes using Student’s *t* test. Insulin resistance was associated with an increase of ≈0.55 to ≈1.0 in aPWV using the HOMA-IR and TyG index, respectively, in both the total sample and stratified by sex. In addition, insulin resistance, as assessed by HOMA-IR and QUICKI, was also associated with AIx@75, with increases ranging from ≈3.8 to ≈5.3% in the total population, with similar values when stratified by sex. Finally, arterial stiffness, as measured by the TyG index, was associated with CAVI with an increase of ≈0.5 m/s stratified and not stratified by sex.

Table 4 summarises the association of aPWV, AIx@75 and CAVI with insulin resistance as determined by HOMA-IR, QUICKI and TyG index by ANCOVA. In adjusted models, insulin resistance determined by HOMA-IR and QUICKI was associated with an increase in aPWV of 0.179 m/s and 0.156 m/s, respectively (*p* = 0.001 and *p* = 0.011). According to sex, men tended to have a greater increase in aPWV than women. In addition, insulin resistance as measured by HOMA-IR and QUICKI was associated with an increase in AIx@75 of 4.17% (*p* = 0.009) and 5.39% (*p* = 0.003), respectively. However, no parameter of insulin resistance was significantly associated with CAVI.

## 4. Discussion

### 4.1. Main Findings

This study examined the association between insulin resistance and markers of arterial stiffness in a population with no prior diagnosis of diseases whose treatment could modify arterial stiffness. In the unadjusted model, insulin resistance increased aPWV by 0.549 to 0.879 m/s and AIx@75 by 3.85 to 5.26%. In adjusted models, this association held for insulin resistance as determined by HOMA-IR and QUICKI, with a slightly reduced effect size for aPWV. However, CAVI was not significantly associated with insulin resistance.

### 4.2. Interpretation

In adjusted models, insulin resistance as assessed by HOMA-IR, and QUICKI was associated with an increase in aPWV of approximately 0.16–0.18 m/s, with a slightly higher trend in men. Similarly, an association with AIx@75 was observed in the total population. In the latter case, the statistical significance was lost when stratifying by sex. This could be due to insufficient sample size, given the high standard deviation of AIx@75. However, the results using the HOMA-IR and QUICKI were similar, even though the cut-off points used estimated a different number of participants with insulin resistance. This is due to two factors: (i) the linearity of insulin resistance (as a continuous variable) with arterial stiffness, so that the cut-offs, although not exact in our population, may be valid for statistical purposes; and (ii) the slightly different values in women compared to men could be due to physiological factors, as well as the use of the same cut-off for both sexes.

Although it may be surprising that insulin resistance as measured by HOMA-IR and QUICKI, but not by the TyG index, was associated with arterial stiffness in the adjusted models, this may be explained by age dependence. The data not included showed a statistically significant Pearson correlation between age and triglycerides and glucose of 0.20 and 0.47, respectively, while for insulin, it was 0.04. In turn, age had a Pearson correlation with HOMA-IR and QUICKI of 0.11, while with the TyG index, it was 0.34. Thus, when no covariate was included, the association was 0.839 m/s (*p* < 0.001); when age was included, it was reduced to 0.129 m/s (*p* = 0.031); and when the other covariates were included, the association was lost.

The study design did not allow causality to be established, but it is reasonable to assume that insulin resistance increases arterial stiffness without ruling out a vicious circle. This can be explained by some pathophysiological mechanisms. As mentioned above, insulin increases nitric oxide synthesis in endothelial cells, leading to vasodilation. However, in states of insulin resistance, it can cause vasoconstriction [11,27,28]. In addition, insulin can activate endothelial cell Na^+^ channels, which together with aldosterone have an additive effect, causing vascular remodelling and stiffening. Additionally, insulin resistance increases the M1 proinflammatory response of macrophages, even increasing the secretion of TNF-α and other cytokines that can worsen vascular function, and decreases the M2 anti-inflammatory response by reducing the synthesis of IL-10 [29,30,31]. Finally, a paradoxical association may occur in early insulin resistance, with a reduction in basal arterial stiffness due to compensatory mechanisms involving increased nitric oxide and increased activity of type 2 angiotensin II receptors. However, persistent insulin resistance over time depletes these compensatory mechanisms and increases arterial stiffness [32].

Our study has two important implications. First, the EVasCu study examined the influence of the relationship between metabolic and cardiovascular health in a healthy population by analysing the association between insulin resistance and arterial stiffness in a healthy population, both risk factors for cardiovascular disease. Due to the design of the study, a confounding or mediating effect of any of the variables cannot be excluded. However, it can be hypothesised that arterial stiffness is, at least in part, a consequence of insulin resistance. With caution, this could explain why some antidiabetic drugs reduce insulin resistance and arterial stiffness [33,34]. This could be a future line of research. Therefore, controlling insulin resistance could reduce arterial stiffness. This is a relationship that has hardly been studied in a healthy population, and other designs are needed to confirm this hypothesis. Second, a 1 m/s increase in PWV is associated with a 15% increase in cardiovascular mortality [35]. Although our results estimate an increase in PWV of 0.16 to 0.31 m/s (depending on the stratification by sex and use of HOMA-IR or QUICKI), these are noteworthy values, as these were presumably healthy populations. In addition, insulin resistance increases the risk of other diseases, which in turn increase mortality [5,6]. Therefore, controlling insulin resistance could prevent premature morbidity and mortality in this population.

### 4.3. Limitations

Our study has some limitations that need to be considered. First, the main limitation was its cross-sectional design, which does not allow us to establish causal relationships and to assume that insulin resistance precedes arterial stiffness, but a vicious circle cannot be ruled out. Second, there may be unstudied mediators that are beyond the scope of this study that might have influenced these associations. Third, the sample size was large enough to detect most of the possible associations. However, it is likely that a larger sample was needed for AIx@75, given some of the observed trends and the high standard deviation. Fourth, although the estimation of aPWV by non-invasive techniques such as Mobil-O-Graph is reliable compared to invasive techniques (gold standard) [36], there could be some differences. Fifth, some self-reported variables, such as smoking, need to be considered with caution, i.e., there is a low proportion of smokers compared to what might be expected. Sixth, due to the design and aim of the study, no specific cut-offs were calculated for our population, but external cut-offs were used. Furthermore, the cut-offs were not stratified by sex, as each cut-off has a different sensitivity and specificity. Seventh, participants with chronic diseases whose disease or treatment could modify arterial stiffness or insulin resistance (i.e., diabetes mellitus, dyslipidaemia, autoimmune diseases, etc.) were excluded from the analyses. However, other types of medication commonly used in acute processes were not considered. Eighth, the study sample included only a Spanish population, so caution is necessary when extrapolating these data to other subjects, age ranges, or non-Caucasian origins.

## 5. Conclusions

Our study reported a strong association between insulin resistance and arterial stiffness, which persisted in adjusted models using HOMA-IR and QUICKI as markers of insulin resistance. However, using the TyG index, no association was observed in adjusted models, perhaps because of its high age dependence. Although causality cannot be established, it is reasonable to postulate that insulin resistance increases arterial stiffness, even in healthy individuals, because insulin resistance increases vasoconstriction and decreases vasodilatory factors, increasing the proinflammatory state of the vasculature and activating Na+ channels in endothelial cells. Therefore, controlling the insulin-resistant population could prevent future morbidity and mortality.

## Figures and Tables

**Table 1:** Baseline characteristics of the participants.

	Total	Men	Women	*p*-Value
Sample	390	144	246	NA
Age	42.05 (13.15)	42.40 (12.47)	41.84 (13.55)	0.688
Physical characteristics				
Weight	70.12 (14.34)	79.82 (12.25)	64.48 (12.33)	<0.001 *
Height (m)	1.68 (0.09)	1.76 (0.07)	1.63 (0.06)	<0.001 *
BMI (kg/m^2^)	24.84 (4.23)	25.63 (3.50)	24.39 (4.54)	0.005 *
Underweight (%)	2.8	0.7	4.1	0.006 *
Normal weight (%)	52.4	45.5	56.5	0.006 *
Overweight (%)	32.1	42.0	26.4	0.006 *
Obesity (%)	12.6	11.9	13.0	0.006 *
Smokers (%)	12.3	9.8	13.8	0.242
Education level (%)				
Illiterate	0	0	0	0.830
No schooling	0	0	0	0.830
Primary school	1.0	0.7	1.2	0.830
Secondary school	11.3	12.6	10.6	0.830
High school	29.8	28.0	30.9	0.830
University degree	56.8	58.0	56.1	0.830
Fasting blood glucose	89.4 (9.9)	91.6 (9.8)	88.2 (9.8)	0.001 *
Fasting insulin	8.51 (6.08)	8.26 (5.45)	8.65 (6.43)	0.538
HOMA-IR	1.94 (1.64)	1.90 (1.34)	1.96 (1.79)	0.754
QUICKI	0.36 (0.03)	0.36 (0.03)	0.36 (0.03)	0.990
TyG Index	8.15 (0.49)	8.23 (0.55)	8.11 (0.44)	0.011 *
Total cholesterol	187.65 (36.22)	185.31 (37.06)	189.01 (35.73)	0.331
LDL-C	118.90 (32.96)	119.77 (33.88)	118.41 (32.47)	0.696
HDL-C	61.62 (13.69)	56.05 (11.95)	64.85 (13.63)	<0.001 *
Triglycerides	87.26 (48.82)	95.13 (61.32)	82.70 (39.22)	0.015 *
Systolic Blood Pressure	116.63 (15.21)	125.04 (12.80)	111.77 (14.37)	<0.001 *
Diastolic Blood Pressure	70.34 (10.59)	72.23 (10.35)	69.24 (10.59)	0.007 *
Mean Arterial Pressure	85.77 (11.39)	89.94 (10.44)	83.42 (11.29)	<0.001 *

Abbreviations: HOMA-IR (Homeostatic Model Assessment for Insulin Resistance); QUICKI (Quantitative Insulin Sensitivity Check Index); TyG Index (Triglycerides-Glucose Index); LDL-C (Low-Density Lipoprotein Cholesterol); HDL-C (High-Density Lipoprotein Cholesterol). Data expressed as mean (standard deviation) or percentage (%). * indicates statistically significant differences (*p* < 0.05).

**Table 2:** Linear regression correlations between markers of arterial stiffness and markers of insulin resistance.

	aPWV			AIx@75			CAVI		
	β	R	*p*-Value	β	R	*p*-Value	β	R	*p*-Value
Total									
HOMA-IR	0.158	0.191	<0.001 *	0.850	0.116	0.023 *	0.009	0.013	0.805
QUICKI	−9.321	0.236	<0.001 *	−66.337	0.191	<0.001 *	−2.014	0.060	0.243
TyG Index	1.042	0.374	<0.001 *	2.624	0.106	0.036 *	2.182	0.251	<0.001 *
Men									
HOMA-IR	0.298	0.298	<0.001 *	1.398	0.177	0.037 *	0.107	0.111	0.190
QUICKI	−11.094	0.288	0.001 *	−62.694	0.205	0.015 *	−6.357	0.172	0.042 *
TyG Index	0.863	0.352	<0.001 *	3.295	0.169	0.044 *	0.554	0.237	0.004 *
Women									
HOMA-IR	0.115	0.151	0.019 *	0.606	0.099	0.125	−0.021	0.035	0.585
QUICKI	−8.267	0.209	0.001 *	−68.641	0.215	0.001 *	0.562	0.018	0.780
TyG Index	1.162	0.379	<0.001 *	4.721	0.190	0.003 *	0.590	0.243	<0.001 *

Abbreviations: HOMA-IR (Homeostatic Model Assessment for Insulin Resistance); QUICKI (Quantitative Insulin Sensitivity Check Index); TyG Index (Triglycerides-Glucose Index); aPWV (aortic Pulse Wave Velocity); AIx@75 (Augmentation index values adjusted to a heart rate of 75 beats per minute); CAVI (Cardio-Ankle Vascular Index). * indicates statistically significant differences (*p* < 0.05).

**Table 3:** Markers of arterial stiffness and presence of insulin resistance by Student’s *t* test.

	aPWV			AIx@75			CAVI		
	Resistance	No Resistance	*p*-Value	Resistance	No Resistance	*p*-Value	Resistance	No Resistance	*p*-Value
Total									
HOMA-IR	6.730 ± 1.506, N = 115	6.181 ± 1.259, N = 267	<0.001 *	19.46 ± 11.14, N = 115	15.62 ± 12.16, N = 267	0.004 *	7.100 ± 1.336, N = 115	7.045 ± 1.079, N = 267	0.669
QUICKI	6.888 ± 1.489, N = 73	6.219 ± 1.298, N = 309	<0.001 *	21.03 ± 11.74, N = 73	15.77 ± 11.83, N = 309	0.001 *	7.119 ± 1.355, N = 73	7.062 ± 1.161, N = 309	0.634
TyG Index	6.932 ± 1.272, N = 129	6.053 ± 1.302, N = 260	<0.001 *	17.38 ± 12.11, N = 129	16.50 ± 11.95, N = 260	0.495	7.420 ± 1.135, N = 129	6.879 ± 1.128, N = 260	<0.001 *
Men									
HOMA-IR	7.048 ± 1.554, N = 42	6.329 ± 1.186, N = 98	0.003 *	13.24 ± 10.01, N = 42	8.71 ± 10.64, N = 98	0.020 *	7.535 ± 1.381, N = 42	7.101 ± 1.231, N = 98	0.068
QUICKI	7.243 ± 1.436, N = 28	6.370 ± 1.266, N = 112	0.002 *	13.07 ± 10.73, N = 28	9.32 ± 10.52, N = 112	0.095	7.557 ± 1.464, N = 28	7.149 ± 1.234, N = 112	0.134
TyG Index	6.925 ± 1.254, N = 57	6.273 ± 1.341, N = 86	0.004 *	11.28 ± 10.34, N = 57	9.26 ± 10.89, N = 86	0.269	7.491 ± 1.206, N = 57	7.054 ± 1.303, N = 86	0.045 *
Women									
HOMA-IR	6.548 ± 1.458, N = 73	6.096 ± 1.296, N = 169	0.017 *	23.04 ± 10.19, N = 73	19.62 ± 11.17, N = 169	0.026 *	6.851 ± 1.252, N = 73	7.013 ± 0.983, N = 169	0.281
QUICKI	6.667 ± 1.494, N = 45	6.133 ± 1.311, N = 197	0.017 *	25.98 ± 9.47, N = 45	19.44 ± 10.96, N = 197	<0.001 *	6.848 ± 1.221, N = 45	6.990 ± 1.035, N = 197	0.421
TyG Index	6.938 ± 1.295, N = 72	5.944 ± 1.273, N = 174	<0.001 *	22.21 ± 11.25, N = 72	20.07 ± 10.79, N = 174	0.165	7.364 ± 1.081, N = 72	6.793 ± 1.024, N = 174	<0.001 *

Abbreviations: HOMA-IR (Homeostatic Model Assessment for Insulin Resistance); QUICKI (Quantitative Insulin Sensitivity Check Index); TyG Index (Triglycerides-Glucose Index); aPWV (aortic Pulse Wave Velocity); AIx@75 (Augmentation index values adjusted to a heart rate of 75 beats per minute); CAVI (Cardio-Ankle Vascular Index). * indicates statistically significant differences (*p* < 0.05).

**Table 4:** Adjusted differences in arterial stiffness markers and the presence of insulin resistance by ANCOVAs.

	aPWV				AIx@75				CAVI			
	Model 1		Model 2		Model 1		Model 2		Model 1		Model 2	
	B	*p*-Value	B	*p*-Value	B	*p*-Value	B	*p*-Value	B	*p*-Value	B	*p*-Value
Total												
HOMA-IR	0.549	<0.001 *	0.179	0.001 *	3.84	0.004 *	4.17	0.009 *	0.055	0.669	0.070	0.537
QUICKI	0.669	<0.001 *	0.156	0.011 *	5.26	0.001 *	5.39	0.003 *	0.072	0.634	−0.058	0.651
TyG Index	0.879	<0.001 *	−0.004	0.947	0.88	0.495	−1.22	0.536	0.541	<0.001 *	0.157	0.258
Men												
HOMA-IR	0.719	0.003 *	0.299	0.001 *	4.52	0.020 *	3.07	0.162	0.434	0.068	0.325	0.137
QUICKI	0.873	0.002 *	0.305	0.007 *	3.75	0.095	1.12	0.669	0.408	0.134	0.126	0.629
TyG Index	0.651	0.004 *	-0.011	0.923	2.03	0.269	−0.14	0.957	0.438	0.045 *	0.296	0.237
Women												
HOMA-IR	0.452	0.017 *	0.129	0.062	3.42	0.026 *	0.664	0.716	−0.162	0.281	0.011	0.934
QUICKI	0.534	0.017 *	0.115	0.127	6.54	<0.001 *	3.896	0.050*	−0.143	0.421	−0.103	0.464
TyG Index	0.994	<0.001 *	−0.047	0.606	2.13	0.165	−4.86	0.044*	0.570	<0.001 *	0.163	0.340

Abbreviations: HOMA-IR (Homeostatic Model Assessment for Insulin Resistance); QUICKI (Quantitative Insulin Sensitivity Check Index); TyG Index (Triglycerides-Glucose Index); aPWV (aortic Pulse Wave Velocity); AIx@75 (Augmentation index values adjusted to a heart rate of 75 beats per minute); CAVI (Cardio-Ankle Vascular Index). * indicates statistically significant differences (*p* < 0.05).

## Data Availability

Data are available upon reasonable request from the corresponding author. The data are not publicly available due to they are original data from our own research project.

## Supplementary Materials

The following supporting information can be downloaded at: https://www.mdpi.com/article/10.3390/nu16060791/s1, Table S1. Correlation coefficients between different markers of insulin resistance. Table S2. Correlation coefficients between different markers of arterial stiffness.

## References

[1] Lebovitz H.E. (2001). Insulin Resistance: Definition and Consequences. Exp. Clin. Endocrinol. Diabetes.

[2] Fahed M., Abou Jaoudeh M.G., Merhi S., Mosleh J.M.B., Ghadieh R., Al Hayek S., El Hayek Fares J.E. (2020). Evaluation of Risk Factors for Insulin Resistance: A Cross Sectional Study among Employees at a Private University in Lebanon. BMC Endocr. Disord..

[3] Friedrich N., Thuesen B., Jørgensen T., Juul A., Spielhagen C., Wallaschofksi H., Linneberg A. (2012). The Association between IGF-I and Insulin Resistance: A General Population Study in Danish Adults. Diabetes Care.

[4] Siri-Tarino P.W., Krauss R.M. (2016). Diet, Lipids, and Cardiovascular Disease. Curr. Opin. Lipidol..

[5] Furie K., Inzucchi S.E. (2008). Diabetes Mellitus, Insulin Resistance, Hyperglycemia, and Stroke. Curr. Neurol. Neurosci. Rep..

[6] Laakso M., Kuusisto J. (2014). Insulin Resistance and Hyperglycaemia in Cardiovascular Disease Development. Nat. Rev. Endocrinol..

[7] Zheng Y., Yin G., Chen F., Lin L., Chen Y. (2022). Evaluation of Triglyceride Glucose Index and Homeostasis Model of Insulin Resistance in Patients with Polycystic Ovary Syndrome. Int. J. Womens Health.

[8] Boutouyrie P., Vermersch S., Laurent S., Briet M. (2008). Cardiovascular Risk Assessment through Target Organ Damage: Role of Carotid to Femoral Pulse Wave Velocity. Clin. Exp. Pharmacol. Physiol..

[9] Zoungas S., Asmar R.P. (2007). Arterial Stiffness and Cardiovascular Outcome. Clin. Exp. Pharmacol. Physiol..

[10] Muniyappa R., Sowers J.R. (2013). Role of Insulin Resistance in Endothelial Dysfunction. Rev. Endocr. Metab. Disord..

[11] Hill M.A., Yang Y., Zhang L., Sun Z., Jia G., Parrish A.R., Sowers J.R. (2021). Insulin Resistance, Cardiovascular Stiffening and Cardiovascular Disease. Metabolism.

[12] Lacolley P., Regnault V., Laurent S. (2020). Mechanisms of Arterial Stiffening. Arterioscler. Thromb. Vasc. Biol..

[13] Weiss W., Gohlisch C., Harsch-Gladisch C., Tölle M., Zidek W., van der Giet M. (2012). Oscillometric Estimation of Central Blood Pressure: Validation of the Mobil-O-Graph in Comparison with the SphygmoCor Device. Blood Press. Monit..

[14] Ji H., Xiong J., Yu S., Chi C., Bai B., Teliewubai J., Lu Y., Zhang Y., Xu Y. (2018). Measuring the Carotid to Femoral Pulse Wave Velocity (Cf-PWV) to Evaluate Arterial Stiffness. J. Vis. Exp..

[15] Santos L.M.D., Gomes I.C., Pinho J.F., Neves-Alves C.M., Magalhães G.S., Campagnole-Santos M.J., da Glória Rodrigues-Machado M. (2021). Predictors and Reference Equations for Augmentation Index, an Arterial Stiffness Marker, in Healthy Children and Adolescents. Clinics.

[16] Perrault R., Omelchenko A., Taylor C.G., Zahradka P. (2019). Establishing the Interchangeability of Arterial Stiffness but Not Endothelial Function Parameters in Healthy Individuals. BMC Cardiovasc. Disord..

[17] Segers P., Rietzschel E.R., Chirinos J.A. (2020). How to Measure Arterial Stiffness in Humans. Arterioscler. Thromb. Vasc. Biol..

[18] Hu H., Cui H., Han W., Ye L., Qiu W., Yang H., Zhang C., Guo X., Mao G. (2013). A Cutoff Point for Arterial Stiffness Using the Cardio-Ankle Vascular Index Based on Carotid Arteriosclerosis. Hypertens. Res..

[19] Poon A.K., Meyer M.L., Tanaka H., Selvin E., Pankow J., Zeng D., Loehr L., Knowles J.W., Rosamond W., Heiss G. (2020). Association of insulin resistance, from mid-life to late-life, with aortic stiffness in late-life: The Atherosclerosis Risk in Communities Study. Cardiovasc. Diabetol..

[20] Saz-Lara A., Cavero-Redondo I., Pascual-Morena C., Martínez-García I., Rodríguez-Gutiérrez E., Lucerón-Lucas-Torres M., Bizzozero-Peroni B., Moreno-Herráiz N., Martínez-Rodrigo A. (2023). Early Vascular Aging as an Index of Cardiovascular Risk in Healthy Adults: Confirmatory Factor Analysis from the EVasCu Study. Cardiovasc. Diabetol..

[21] Henríquez S., Jara N., Bunout D., Hirsch S., de la Maza M.P., Leiva L., Barrera G. (2013). Variability of Formulas to Assess Insulin Sensitivity and Their Association with the Matsuda Index. Nutr. Hosp..

[22] Alizargar J., Bai C.-H., Hsieh N.-C., Wu S.-F.V. (2020). Use of the Triglyceride-Glucose Index (TyG) in Cardiovascular Disease Patients. Cardiovasc. Diabetol..

[23] Arama V., Tiliscan C., Streinu-Cercel A., Ion D., Mihailescu R., Munteanu D., Hristea A., Arama S.S. (2013). Insulin Resistance and Adipokines Serum Levels in a Caucasian Cohort of Hiv-Positive Patients Undergoing Antiretroviral Therapy: A Cross Sectional Study. BMC Endocr. Disord..

[24] Gayoso-Diz P., Otero-González A., Rodriguez-Alvarez M.X., Gude F., García F., De Francisco A., Quintela A.G. (2013). Insulin Resistance (HOMA-IR) Cut-off Values and the Metabolic Syndrome in a General Adult Population: Effect of Gender and Age: EPIRCE Cross-Sectional Study. BMC Endocr. Disord..

[25] García-Quismondo Á., Del Cañizo F.J., Dorado J., Sánchez-Muniz F.J. (2017). Classical and Emergent Cardiovascular Disease Risk Factors in Type 2 Diabetics from the Vallecas Area (DICARIVA Study). Nutr. Hosp..

[26] Weir C.B., Jan A. (2023). BMI Classification Percentile and Cut Off Points. StatPearls.

[27] Isenovic E.R., Divald A., Milivojevic N., Grgurevic T., Fisher S.E., Sowers J.R. (2003). Interactive Effects of Insulin-like Growth Factor-1 and Beta-Estradiol on Endothelial Nitric Oxide Synthase Activity in Rat Aortic Endothelial Cells. Metabolism.

[28] Muniyappa R., Sowers J.R. (2012). Endothelial Insulin and IGF-1 Receptors: When Yes Means NO. Diabetes.

[29] Jia G., Aroor A.R., DeMarco V.G., Martinez-Lemus L.A., Meininger G.A., Sowers J.R. (2015). Vascular Stiffness in Insulin Resistance and Obesity. Front. Physiol..

[30] Aroor A.R., Demarco V.G., Jia G., Sun Z., Nistala R., Meininger G.A., Sowers J.R. (2013). The Role of Tissue Renin-Angiotensin-Aldosterone System in the Development of Endothelial Dysfunction and Arterial Stiffness. Front Endocrinol.

[31] Aroor A.R., McKarns S., Demarco V.G., Jia G., Sowers J.R. (2013). Maladaptive Immune and Inflammatory Pathways Lead to Cardiovascular Insulin Resistance. Metabolism.

[32] Brillante D.G., O’Sullivan A.J., Howes L.G. (2009). Arterial Stiffness in Insulin Resistance: The Role of Nitric Oxide and Angiotensin II Receptors. Vasc. Health Risk Manag..

[33] Wang J., Wang Y., Wang Y., Li Y., Zhang J., Zhang H., Fu X., Guo Z., Yang Y., Kang K. (2023). Effects of first-line antidiabetic drugs on the improvement of arterial stiffness: A Bayesian network meta-analysis. J. Diabetes.

[34] Lyu Y.S., Hong S., Lee S.E., Cho B.Y., Park C.-Y. (2024). Efficacy and safety of enavogliflozin vs. dapagliflozin as add-on therapy in patients with type 2 diabetes mellitus based on renal function: A pooled analysis of two randomized controlled trials. Cardiovasc. Diabetol..

[35] Ben-Shlomo Y., Spears M., Boustred C., May M., Anderson S.G., Benjamin E.J., Boutouyrie P., Cameron J., Chen C.-H., Cruickshank J.K. (2014). Aortic Pulse Wave Velocity Improves Cardiovascular Event Prediction: An Individual Participant Meta-Analysis of Prospective Observational Data from 17,635 Subjects. J. Am. Coll. Cardiol..

[36] Walser M., Schlichtiger J., Dalla-Pozza R., Mandilaras G., Tengler A., Ulrich S., Oberhoffer F.S., Oberhoffer-Fritz R., Böhm B., Haas N.A. (2023). Oscillometric pulse wave velocity estimated via the Mobil-O-Graph shows excellent accuracy in children, adolescents and young adults: An invasive validation study. J. Hypertens..

