# Supplementary material for: Exploring the Influence of Insulin Resistance on Arterial Stiffness in Healthy Adults: From the Metabolic and Cardiovascular Health Insights of the EVasCu Study
Source: Nutrients. 2024 Mar 11;16(6):791. doi: 10.3390/nu16060791 (PMC10974428; doi:10.3390/nu16060791)
Supplement: Supplementary file 1 [file nutrients-16-00791-s001.zip › nutrients-2901532-supplementary.pdf]

Supplementary material

Exploring the influence of insulin resistance on arterial stiffness in healthy adults: from the metabolic and cardiovascular health insights of the EVasCu study

Nutrients

Index

Table S1. Correlation coefficients between different markers of insulin resistance.

Table S2. Correlation coefficients between different markers of arterial stiffness.

Table S1. Correlation coefficients between different markers of insulin resistance.

Dependent vs independent variable	Correlation
HOMA-IR vs QUICKI	R = 0.807, $p < 0.001$
HOMA-IR vs TyG Index	R = 0.427, $p < 0.001$
QUICKI vs TyG Index	R = 0.491, $p < 0.001$

Table S2. Correlation coefficients between different markers of arterial stiffness.

Dependent vs independent variable	Correlation
aPWV vs AIx@75	R = 0.190, $p < 0.001$
aPWV vs CAVI	R = 0.627, $p < 0.001$
CAVI vs AIx@75	R = 0.115, $p = 0.027$
